# Length effects on the dynamic process of cellular uptake and exocytosis of single-walled carbon nanotubes in murine macrophage cells

**DOI:** 10.1038/s41598-017-01746-9

**Published:** 2017-05-10

**Authors:** Xuejing Cui, Bin Wan, Yu Yang, Xiaomin Ren, Liang-Hong Guo

**Affiliations:** 1 State Key Laboratory of Environmental Chemistry and Ecotoxicology, Research Center for Eco-Environmental Sciences, Chinese Academy of Sciences, Beijing, 100085 China; 2 University of Chinese Academy of Sciences, Beijing, 100049 China; 3 Institute of Environment and Health, Jianghan University, Wuhan, Hubei 430056 China

## Abstract

Cellular uptake and exocytosis of SWCNTs are fundamental processes determining their intracellular concentration and effects. Despite the great potential of acid-oxidized SWCNTs in biomedical field, understanding of the influencing factors on these processes needs to be deepened. Here, we quantitatively investigated uptake and exocytosis of SWCNTs in three lengths-630 (±171) nm (L-SWCNTs), 390 (±50) nm (M-SWCNTs), and 195 (±63) nm (S-MWCNTs) in macrophages. The results showed that the cellular accumulation of SWCNTs was a length-independent process and non-monotonic in time, with the most SWCNTs (3950 fg/cell) accumulated at 8 h and then intracellular SWCNTs dropped obviously with time. The uptake rate of SWCNTs decreased with increasing concentration, suggesting that intracellular SWCNTs accumulation is a saturable process. After refreshing culture medium, we found increasing SWCNTs in supernatant and decreasing intracellular SWCNTs over time, confirming the exocytosis occurred. Selective inhibition of endocytosis pathways showed that the internalization of SWCNTs involves several pathways, in the order of macropinocytosis> caveolae-mediated endocytosis> clathrin-dependent endocytosis. Intriguingly, clathrin-mediated endocytosis is relatively important for internalizing shorter SWCNTs. The dynamic processes of SWCNTs uptake and exocytosis and the mechanisms revealed by this study may render a better understanding on SWCNT toxicity and facilitate the design of CNT products with mitigated toxicity and desired functions.

## Introduction

Carbon nanotubes (CNTs), due to their high specific surface area, strength, conductivity, magnetic susceptibility and catalytic activity, have become one of the most shining stars among nanomaterials and are undergoing rapid pace of development. CNTs possess great potentials in both consumer and industrial applications such as electronics, coatings, super capacitors, thermoplastics, and batteries^1–5^. Additionally, CNTs are also used in pharmaceutical/biomedical devices for bone grafting, tissue repair, drug delivery, and disease diagnostics^6, 7^. The increasing production and use of CNTs will inevitably increase human exposure, raising concerns for their potential health risks, which has to be addressed for a sustainable development of nanotechnologies.

CNTs could enter organisms *via* inhalation, digestion or injection of therapeutic materials^8, 9^ and readily translocate among tissues and organs through bloodstream^10, 11^. Exposure of CNTs exerts negative effects on the normal physiological functions of respiratory system, cardiovascular system, and immune system, inducing inflammation, granulomas and sub-pleural fibrosis^12–14^, progression of atherosclerosis^15, 16^, and allergic immune responses^17^. The highly phagocytic macrophages represent the first line of defense against pathogens and particles and are one of the major cell types involved in uptake and clearance of these particles^18^. They are well recognized as a major source of pro-inflammatory and fibrogenic mediators, including cytokines, growth factors, and reactive oxygen species (ROS)^19^. Recently, we have demonstrated that single-walled carbon nanotubes (SWCNTs) could readily enter macrophages and interfere with the biogenesis and gene expression of various organelles, inducing inflammatory response and impairing macrophage functionality^20, 21^.

Several physicochemical characteristics including nano-structure, composition, surface charge, agglomeration state and aspect ratio were shown to influence the bioactivity of SWCNTs^22^. The high aspect ratio of CNTs is comparable to that of pathogenic fibers such as asbestos, which were shown to cause length-dependent toxicity^23, 24^. A number of *in vivo* and *in vitro* studies showed that longer CNTs had higher potential in inducing lung fibrogenic and inflammatory responses including oxidative stress, collagen expression, transforming growth factor-beta production and cell apoptosis^19, 25^. This is probably due to the frustrated phagocytosis of longer fibers by cells, leading to continuous production of ROS and enhanced toxicity^26, 27^. In addition, fiber length also influences CNTs retention and clearance from the lungs^28, 29^ and the relationship between CNT length and disease progression has been demonstrated as well^23, 28^.

At the cellular level, interactions between cells and CNTs influence the amount of CNTs that are associated with or within cells and thus precede the occurrence of toxicity. Holt *et al*. reported that bovine serum albumin coated SWCNTs (SWCNTs-BSA) had a rapid rate of uptake and relatively slow rate of recovery in embryo fibroblasts (NIH-3T3), with the mass of SWCNTs-BSA per cell reached a steady state within a minute^30^. By comparing the kinetics of SWCNTs-BSA and Pluronic F127 coated SWCNTs (SWCNTs-PF127), Boyer *et al*. demonstrated that uptake of SWCNTs in J774A.1 cells involved both membrane association and receptor mediated internalizations^31^. A length-selective uptake of DNA-wrapped SWCNTs was illustrated in human fibroblasts^32^. Given the significant role of macrophages played in dictating the fate, processing and ultimate effect of CNTs and the length-dependent effects of SWCNTs, however, the information on the rate and mechanism of length-dependent SWCNTs uptake and excretion from cells, especially for macrophages, are very limited. An illustration on these issues would be of great significance for a better understanding on the toxicity of CNTs and facilitate the design of CNT products with desired functionality and lower toxicity.

To this end, we prepared three well-dispersed SWCNTs: long (L-SWCNTs, 630 (±171) nm), medium (M-SWCNTs, 390 (±50) nm), and short (S-SWCNTs, 195 (±63) nm) SWCNTs by controlling acid-oxidation time and quantitatively analyzed the length-dependent effects on the dynamic processes of SWCNTs uptake and exocytosis in macrophages. Specifically, intracellular SWCNTs amount was determined by sodium dodecyl sulfate polyacrylamide gel electrophoresis (SDS-PAGE)^33^ at different time points in macrophages exposed to various concentrations of SWCNTs. Simultaneously, the secretion of SWCNTs was studied by monitoring SWCNTs amount over time in culture medium supernatant using ultraviolet-visible-near infrared (UV-vis-NIR) spectroscopy after the removal of SWCNTs exposure solution. In order to gain insights into the mechanisms of SWCNTs endocytosis, we used four inhibitors, each blocking a specific endocytosis pathway, to pre-treat the cells, which is followed by SWCNTs exposure and intracellular SWCNTs measurement. The results showed an active SWCNTs exocytosis and macropinocytosis being the major mechanism for the endocytosis of all SWCNTs in macrophages.

## Results

### Preparation and characterization of SWCNTs

**Figure 1:**
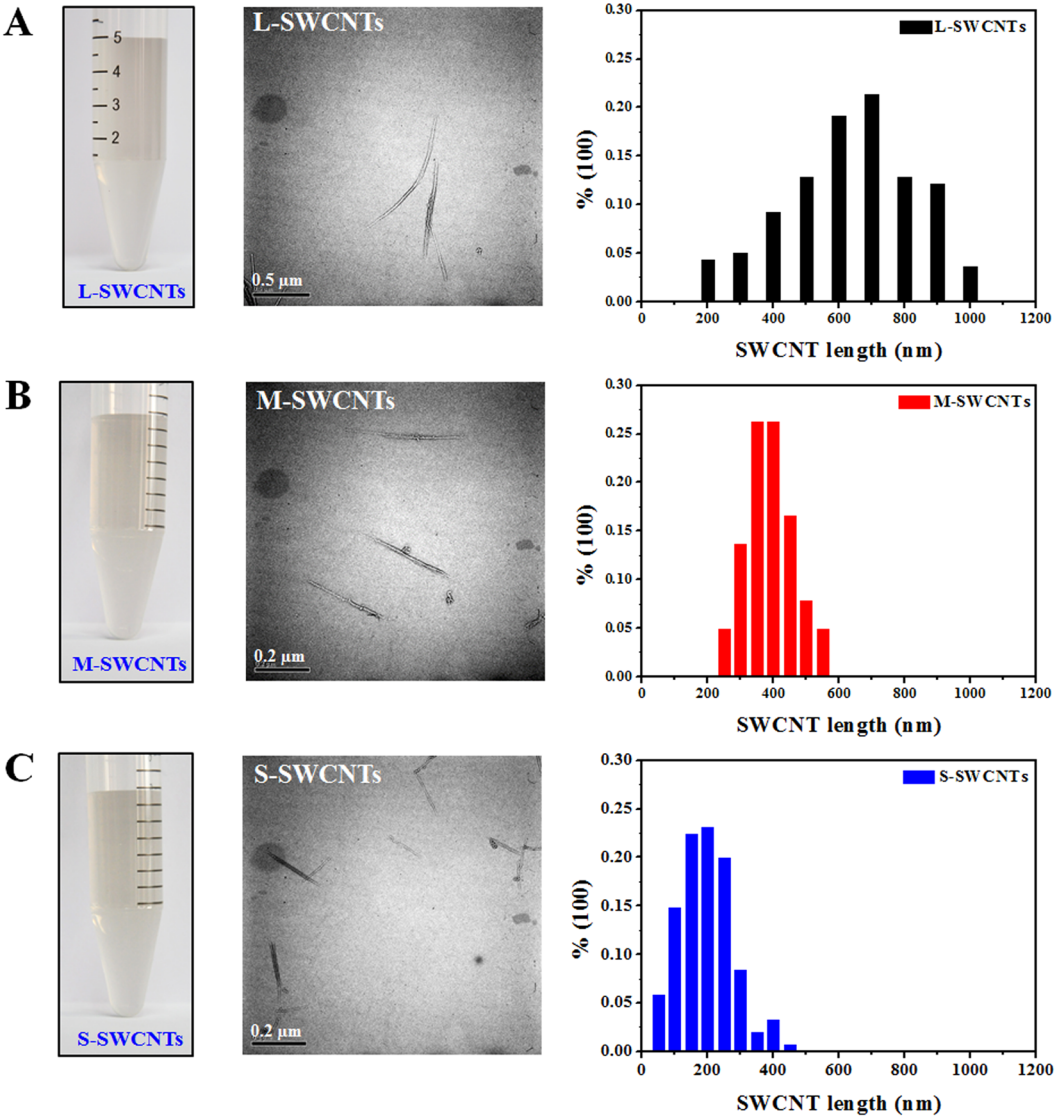
SWCNTs suspensions, TEM images, and length distribution. Stable suspensions(Left), typical TEM image (Middle), and length distribution (Right) of (**A**) L-, (**B**) M-, and (**C**) S-SWCNTs. Length distribution histograms of L-, M-, and S-SWCNTs were generated by measuring approximately 100 individual nanotubes in representative TEM images. In TEM images, the scale bars represent 0.5 µm for L-SWCNTs and 0.2 µm for M and S-SWCNTs.

Three different lengths of SWCNTs were prepared according to the experimental section. The resulting SWCNTs remain well dispersed and stable for months in aqueous media, including water and culture medium (Fig. 1). Characterization by transmission electron microscopy (TEM) showed that the SWCNTs retained the structural integrity of carbon nanotubes (Fig. 1, middle panel). One hundred CNTs of each SWCNT type were analyzed using Image-Pro Plus 6.0 software and the average lengths were found to be 630 (±171) nm (L-SWCNTs), 390 (±50) nm (M-SWCNTs), and 195 (±63) nm (S-SWCNTs) (Fig. 1, right panel). The average length distribution of L-, M, and S-SWCNTs are statistically different (p < 0.05) from each other by ANOVA analysis (one way), and the 95% confidence interval of the mean difference between L- and M-SWCNTs is from 224 to 289 nm, between L- and S-SWCNTs is from 427 to 487 nm, and between M- and S-SWCNTs is from 169 to 233 nm. Fourier-transformed infrared spectroscopy (FT-IR) analyses indicated the presence of carboxyl (ν = 1635 cm^−1^, −COOH) and hydroxyl groups (ν = 1397 cm^−1^, −OH) on the surface of these SWCNTs (Supplementary Fig. 1A). In addition, the hydrodynamic diameters and the zeta potentials of these SWCNTs in water, serum-free medium (SFM), and serum-supplemented medium (SSM) were listed in Table 1. Hydrodynamic diameters (HD) and surface charges of these SWCNTs changed in SFM and SSM compared to water because of charge neutralization in medium, which is in accordance with the results of a study using carboxyl functionalized-SWCNTs^34^. Significant lower HDs of SWCNTs in SSM compared with those in SFM was due to serum proteins facilitating the dispersion of SWCNTs. It is worth of note that, these SWCNTs had similar zeta potentials in a specific medium, suggesting that they have similar surface charges. The average length of SWCNTs were determined by measuring the length of SWCNTs in TEM images, but HDS was measured as hydrodynamic diameters by assuming round shape of tested particles. Since SWCNTs are actually in shape of long fibers, and thus there was inconsistence between HDS and average length. But if the particles were stabilized by cultural media or proteins^35^, we start to see the correlation between HDS and average sizes, as shown in Table 1 (SFM and SSM columns). The inductively coupled plasma mass spectrometry (ICP-MS) measurement detected a negligible iron content of 0.056 wt% for SWCNTs, as reported previously^20^, cobalt and nickel were not detected in the samples. The Raman spectroscopy of SWCNTs revealed minimal amount of radial breathing mode (RBM) intensity above 250 cm^−1^, suggesting minimal SWCNT bundling (Supplementary Fig. 1B).

**Table 1:** Hydrodynamic Size (HDS, nm) and Zeta Potential (ZP, mV) of L, M, and S-SWCNTs in water, serum-free medium (SFM), serum-supplemented medium (SSM).

Samples	Water		SFM		SSM	
	HDS	ZP	HDS	ZP	HDS	ZP
L-SWCNTs	91.1 ± 7.3	−49.4 ± 3.8	1417.0 ± 121	−21.6 ± 1.5	134.1 ± 10.1	−8.2 ± 0.3
M-SWCNTs	71.5 ± 1.2	−47.3 ± 1.5	1258.3 ± 116	−21.2 ± 1.3	118.3 ± 6.2	−8.1 ± 0.4
S-SWCNTs	75.6 ± 4.3	−48.8 ± 1.0	1239.7 ± 109	−19.1 ± 0.9	112.6 ± 3.3	−7.8 ± 0.1

### Cellular uptake of SWCNTs in RAW264.7 cells

**Figure 2:**
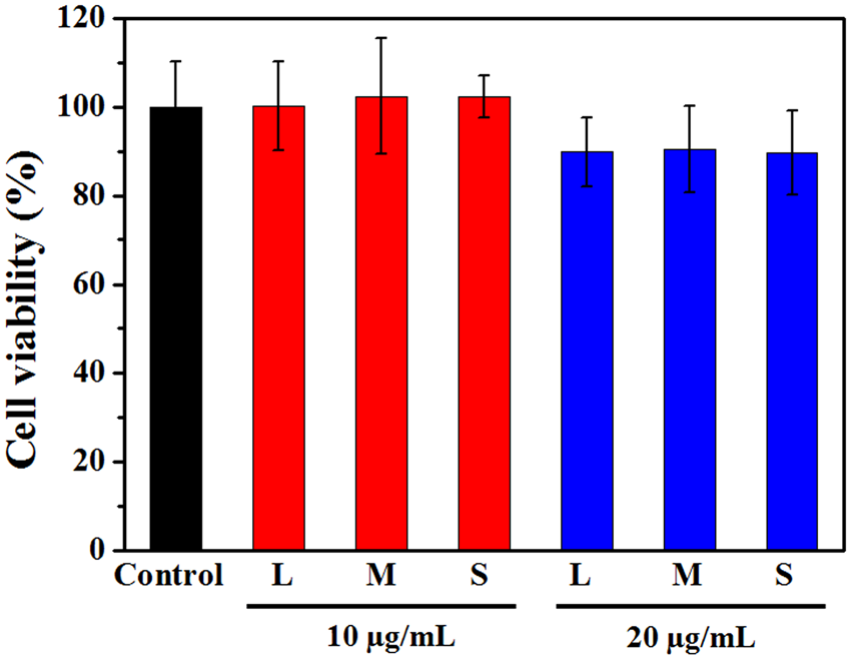
The viability (Treatment/control) of cells treated with different concentrations of L-SWCNTs (L), M-SWCNTs (M), and S-SWCNTs (S) for 24 h, as determined by WST-1 assay.

First, we measured the viability of the cells exposed to 0, 10, and 20 µg/mL SWCNTs by water soluble tetrazolium/formazan (WST-1) method, and the results showed no cell death in 10 µg/mL exposure group and ~90% viability in 20 µg/mL group (Fig. 2). Next, to investigate the SWCNTs endocytosis of RAW264.7 cells, SDS-PAGE was employed to quantify the amount of SWCNTs in cells as described previously^33^. The reliability of SDS-PAGE was verified via Raman spectra scanning and coomassie blue staining of the gel, which showed the separation of cellular proteins from SWCNTs and the deposition of SWCNTs within gel as a band, no SWCNTs were detected in other areas of the gel (Supplementary Fig. 2). The standard curve (R^2^ = 0.992) for protein amount against cell number was acquired by bicinchoninic acid (BCA) assay and the standard curve (R^2^ = 0.987) for the amount of SWCNTs against the band intensity was determined by SDS-PAGE gel analysis, both standard curves showed good linear relationships (Supplementary Fig. 3). Therefore, with known loading amount of protein and SWCNT band intensity, we can calculate the average mass amount of SWCNTs per cell. In addition, we measured the cell proliferation over time (Supplementary Fig. 4), and normalized the SWCNTs amount per cell against cell proliferation rate.

**Figure 3:**
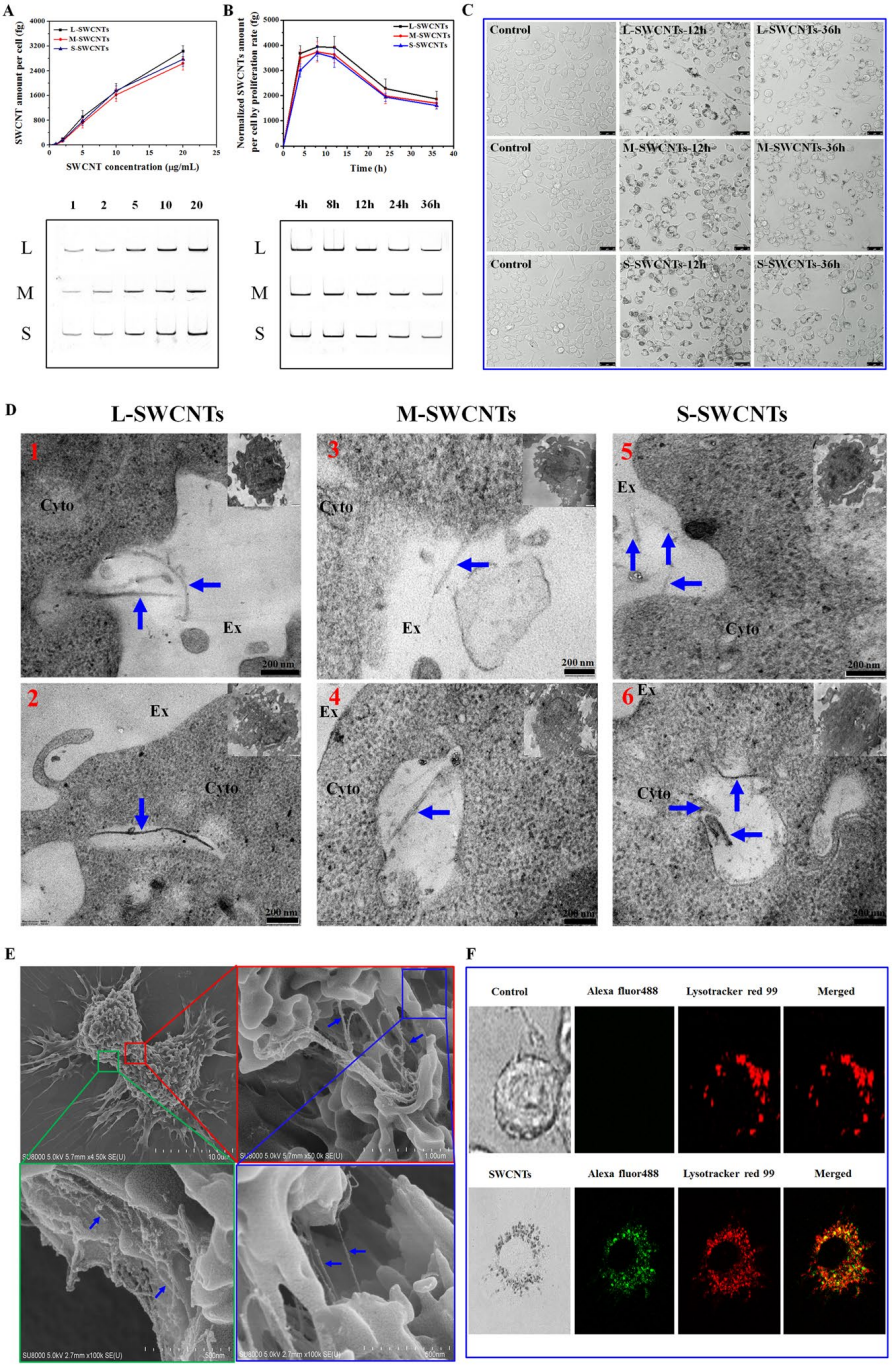
Cellular uptake of SWCNTs in RAW264.7 cells. (**A**) SDS-PAGE gel quantification of concentration-dependent cellular uptake of SWCNTs in macrophages after 24 h exposure. Lower panels show corresponding representative SWCNTs bands in gel. (**B**) Quantification of time-dependent cellular uptake of 10 µg/mL SWCNTs normalized by cell proliferation rate; Lower-panels show corresponding representative SWCNTs bands visualized on gel. (**C**) Selected (low cell density area) bright field images of cells cultured with or without 10 µg/mL SWCNTs for 0, 12 and 36 h to clearly show the changing tendency of SWCNT amount within individual cells. Scale bar = 25 µm. (**D**) Typical TEM images showing different stages of endocytosis process of L-, M-, and S-SWCNTs (blue arrows). Up panels show the initial interactions between SWCNTs and cell membrane (1, 3, 5); down panels show the L-, M-, and S-SWCNTs (blue arrows) entrapped in early endosomes with relatively clean background (2, 4, 6). Ex, Cyto, and MT indicates extracellular space, cytoplasm, and mitochondria, respectively. Scale bar is 200 nm for TEM images. The whole cell was shown as insert on the top right of each image; (**E**) Typical SEM images of L-SWCNTs on cell surface showing the details on the interactions between SWCNTs and cell membrane. (**F**) Co-localization of SWCNTs (labeled with alexa fluor 488, green) and lysosomes (labeled with lysotracker red 99, red) (lower panel). For (**A** and **B**), data are presented as the mean of three independent experiments. Error bars represent the standard error of the mean.

As shown in Fig. 3A, there was a concentration-dependent uptake for all three lengths of SWCNTs, with similar rates. After 24 h exposure, the amount of SWCNTs taken up by cells increased with increasing SWCNTs concentration. Interestingly, the rate of uptake was approximately linear at lower concentrations (1–10 µg/mL), however, the efficiency decreased at higher concentrations (10–20 µg/mL). Then, we further determined the SWCNTs uptake over time in 10 µg/mL exposure group and found that cells took up SWCNTs quickly and reached the highest amount (approximately 3950 fg/cell for all types of SWCNTs) within the first 8 h (Fig. 3B). Surprisingly, the amount of SWCNTs within cells proceeded to decrease after then, dropping to 1936–2281 fg/cell at 24 h and to 1604–1864 fg/cell at 36 h, respectively, even after considering the proliferation of cells (Supplementary Fig. 4). There were no significant differences between the three lengths of SWCNTs. All data suggested that the internalization of these SWCNTs by cells is not a monotonic process. In addition, through bright field imaging, we directly showed that SWCNT within cells tended to decrease after prolonged exposure (12 h vs. 36 h) (Fig. 3C). These data, although limited, partly suggested the occurrence of SWCNT exocytosis.

In addition, TEM images revealed the endocytosis process of L-, M-, and S-SWCNTs, which began with the interaction between SWCNTs and cell membrane (Fig. 3D, up panel, 1, 3, 5), and then entrapped in early endosomes (Fig. 3D, down panel, 2, 4, 6), indicating that SWCNTs were indeed internalized by macrophages. Furthermore, we also observed association of SWCNTs with cell membrane by SEM (Fig. 3E). Moreover, we tracked the specific location of SWCNTs within living cells by labeling SWCNTs with fluorescent Alexa Fluor 488. The successful conjugation of Alexa Fluor 488 on SWCNTs was characterized by FT-IR (Supplementary Fig. 5). As shown in Fig. 3F, the green fluorescence from SWCNT-Alexa Fluor 488 well overlapped with the red signal from Lysotracker Red, a fluorescent probe targeting lysosomes, indicating that SWCNTs were mostly accumulated in lysosomes. These results indicated that accumulation of SWCNTs by cells involved both membrane association processes and internalization, and most SWCNTs were sorted to lysosomes. The length of SWCNTs did not influence the net accumulation of CNTs, which tend to increase at early stage of exposure and then decreased over time.

### Exocytosis and intracellular retention of SWCNTs

**Figure 4:**
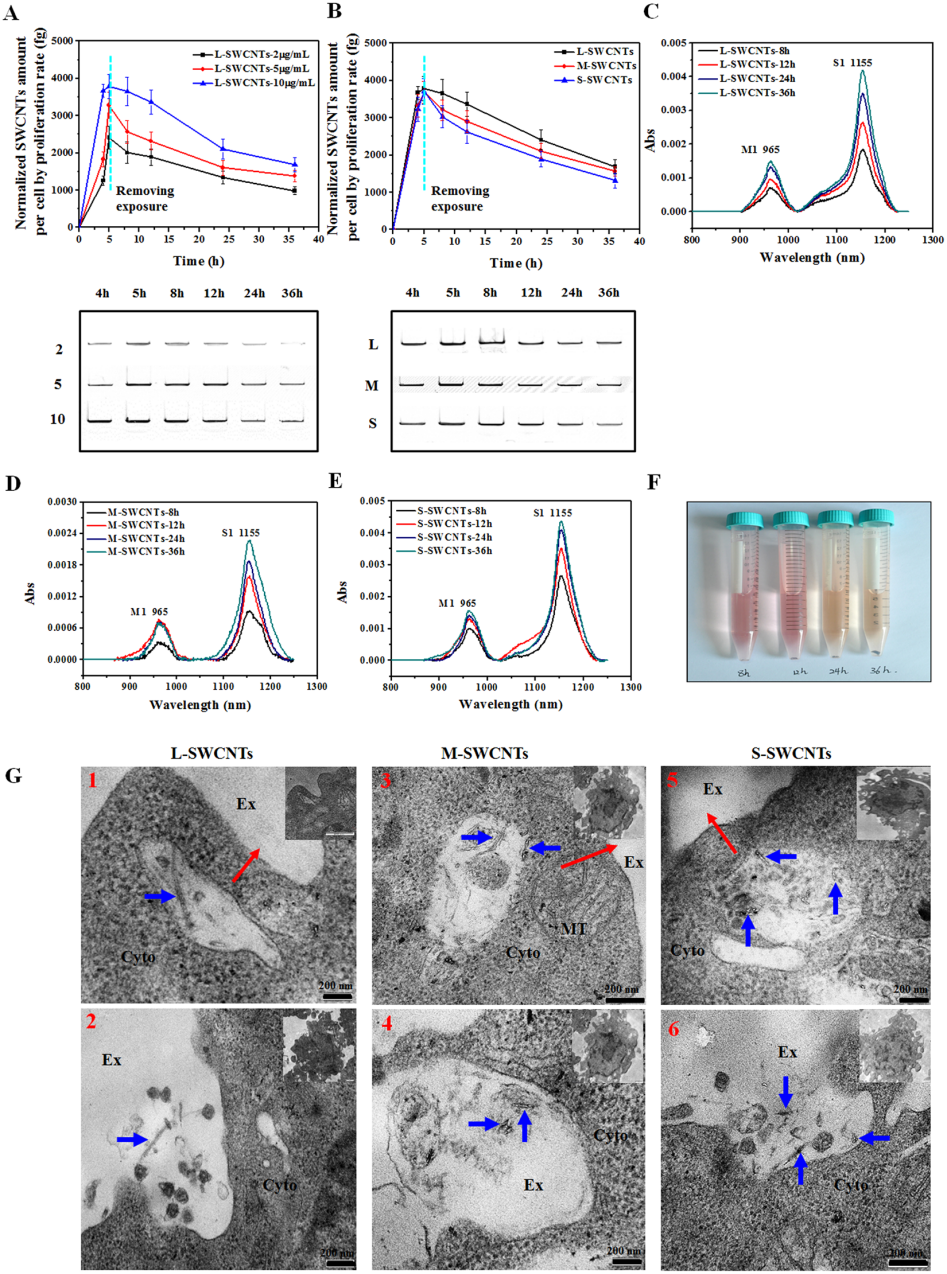
Exocytosis of SWCNTs. (**A**) Normalized measurement of L-SWCNTs by SDS-PAGE gel analyses within cells at different time points after removing exposure solutions at 5 h. (**B**) Normalized measurement of L-, M-, and S-SWCNTs (10 µg/mL) retained in cells at different time points after removing exposure solutions at 5 h. No statistical significant difference was detected among three groups of SWCNTs (p > 0.05). UV-vis-NIR spectra of the supernatant of L-SWCNTs (**C**), M-SWCNTs (**D**), and S-SWCNTs (**E**) at different time points (8–36 h) after replacing SWCNTs exposure solutions with fresh medium (without SWCNTs). (**F**) L-SWCNTs pellets after replacing the SWCNTs exposure solutions with fresh medium and centrifuging the supernatants collected between 8 and 36 h. (**G**) Typical TEM images showing cellular exocytosis process of L-, M-, and S-SWCNTs (blue arrows). Up panels show typical TEM images of SWCNT-containing vesicles (1, 3, 5) near cell membrane and to be released with complicate lysosomal contents to extracellular space, red arrows indicate the postulated direction of vesicles moving toward cell membrane; down panels show the exocytosed SWCNTs along with substantial lysosomal contents (2, 4, 6). Ex, Cyto, and MT indicates extracellular space, cytoplasm, and mitochondria, respectively. Scale bar is 200 nm for TEM images. For (**A** and **B**), data are presented as the mean of three independent experiments. Error bars represent the standard error of mean.

**Figure 5:**
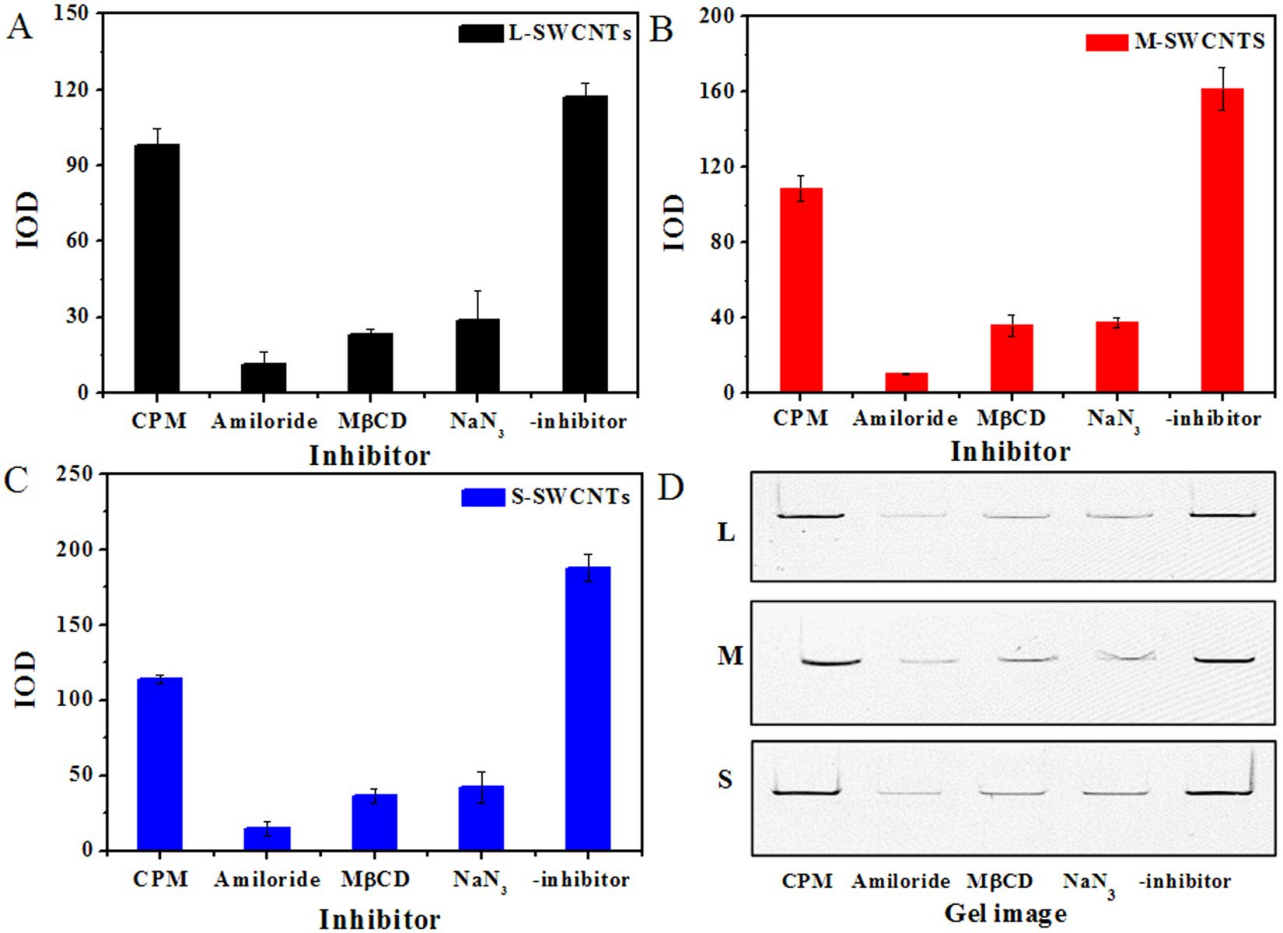
The inhibition of CPM, MβCD, NaN_3_, and amiloride on the endocytosis of L- (**A**), M- (**B**), and S-SWCNTs (**C**). (**D**) Typical SDS-PAGE gel images for intracellular L-, M-, and S-SWCNTs pretreated with inhibitors. The intensity of the dark band (IOD) of the SWCNTs was analyzed using Gelpro software. Data are presented as the means of three independent experiments. Error bars represent the standard error of mean.

The significant reduction in intracellular SWCNTs after prolonged SWCNTs exposure was unexpected and raised the question as to whether the reduction was due to SWCNTs exocytosis or not. To answer the question, we replaced the exposure solution with fresh cultural medium and measured the intracellular SWCNTs. Results from SDS-PAGE gel analyses, after normalizing against cell proliferation, showed that cells, after removing the exposure medium (after 5 h exposure), from the 2, 5, and 10 µg/mL groups repelled about 59%, 57% and 55% of the internalized L-SWCNTs at 36 h, respectively. The remaining SWCNTs retained within cells were 985, 1380, and 1691 fg/cell, respectively (Fig. 4A). For different lengths of SWCNTs, 55% of internalized L-SWCNTs, 57% of M-SWCNTs, and 65% of S-SWCNTs were excreted at 36 h (Fig. 4B). Although the difference in the repelling efficiency is tiny (only ~10%), S-SWCNTs were the most easily expelled, in the order of S-SWCNTs > M-SWCNTs > L-SWCNTs. Since all cells are viable under 10 µg/mL exposure (Fig. 2), we can rule out the possibility that the expelled SWCNTs were due to the cell death. To further confirm that SWCNTs were indeed exocytosed from the cells, the supernatant was analyzed using UV-vis-NIR and measured at 965 nm and 1155 nm, where SWCNT absorption peaked^36, 37^. Results showed that absorption increased over time for L-SWCNTs (Fig. 4C), suggesting the occurrence of SWCNTs exocytosis by cells after the removal of exposure medium. Meanwhile, the cell viability was determined to be over 97% throughout the experiment (0–36 h) by using an automatic cell counter (Countess, Invitrogen) (Supplementary Fig. 6), which rules out the influence of cell death on the release of intracellular SWCNTs. Similar results were observed for M- and S-SWCNTs (Fig. 4D,E). SWCNTs accumulation in the medium from exocytosis was also confirmed by the increasing size of SWCNTs pellets after the centrifugation of culture medium (Fig. 4F). Moreover, the TEM images showed that the ongoing released SWCNTs located in the lysosomes with inner contents near cell membrane (Fig. 4G, 1, 3, 5), and the internalized SWCNTs were exocytosed to extracellular space along with substantial undigested contents (Fig. 4G, 2, 4, 6).

### Cellular uptake mechanism of SWCNTs

To elucidate the cellular uptake mechanism of SWCNTs, we used four inhibitors, including chlorpromazine (CPM, clathrin inhibitor), methyl-β-cylodextrin (MβCD, caveolae inhibitor), sodium azide (NaN_3_, ATP production disruptor), and amiloride (micropinocytosis inhibitor)^38–40^, each blocks a specific endocytosis pathway.

The cytotoxicity of the four inhibitors was firstly evaluated by the WST-1 assay, and results showed negligible cytotoxicity of these inhibitors at given concentrations (Supplementary Fig. 7). The inhibition of SWCNTs endocytosis was calculated by comparing the intracellular SWCNTs of the control group (SWCNTs treatment only) with the treatment groups (SWCNTs and inhibitor treatment). The most efficient inhibitor of SWCNTs uptake was amiloride: the SWCNTs band intensity of cells treated by amiloride was only 6.4–9.5% when compared with the control group, suggesting that macropinocytosis was the main mechanism of SWCNTs internalization. The order of inhibiting effects was amiloride > MβCD > NaN_3_ > CPM (Fig. 5 and Table 2). NaN_3_ treatment resulted in 22.6–25.3% SWCNTs band intensity (Fig. 5 and Table 2), indicating that SWCNT endocytosis was mostly energy dependent and other energy-independent processes, e.g. diffusion, might also play a role in the uptake process of SWCNTs. Similar results were observed for M- (Fig. 5B) and S-SWCNTs (Fig. 5C). Interestingly, the results also showed that CPM had different inhibition efficiencies for different lengths of SWCNTs. For example, CPM treatment resulted in 83.6% SWCNTs band intensity for L-SWCNTs, and the SWCNTs band intensity remarkably decreased to 64.3% and 60.7% for M- and S-SWCNTs (P < 0.05, compared to L-SWCNTs), respectively (Table 2). However, there is no significant difference between M- and S-SWCNTs for CPM treatment. These results indicate that clathrin-mediated endocytosis was length dependent and relatively important for short-length SWCNTs.

**Table 2:** The inhibition of SWCNTs endocytosis by specific inhibitors.

Inhibitors	CPM	Amiloride	MβCD	NaN_3_
L-SWCNTs	83.6% ± 1.1%	9.5% ± 2.2%	20.1% ± 1.1%	25.3% ± 5.8%
M-SWCNTs	64.3% ± 2.0%^a^	6.4% ± 0.1%	22.1% ± 4.6%	22.6% ± 2.4%
S-SWCNTs	60.7% ± 1.6%^a^	7.7% ± 2.0%	19.7% ± 3.1%	23.2% ± 6.6%

The percentages were obtained by normalizing the SWCNTs band intensity of the inhibitor treatment groups against the control group (IOD_inhibitor_/IOD_control_). Data are presented as mean ± standard error of mean.

^a^Statistical evaluation by ANOVA: p < 0.05 compared to L-SWCNTs.

## Discussion

Successful application of SWCNTs most often begins with the control of dispersion because well-dispersed SWCNTs are of great interest for biological applications and full characterization of toxicological effects can only be performed with nanomaterials that are well-dispersed in aqueous phase^41^. Most studies used CNTs modified with polymers or macromolecules^30, 42–44^ because such coating or conjugation greatly increases the stability of CNTs suspension. Alternatively, an acid-oxidation procedure in which SWCNTs react with mixtures of sulphuric acid and nitric acid can greatly facilitate the dispersion of SWCNTs in aqueous environments^45^, enabling new biological and biomedical applications with enormous potentials^46–48^. This type of SWCNTs has been used to develop tumor-targetable multifunctional SWCNT platforms^49^. The oxidation and subsequent filtration removes the most majority of metal impurities. By controlling the oxidation time, we successfully obtained three types of SWCNTs varying in their average lengths as reported previously^46, 50^ but with similar surface charges (Table 1). The concentrations used in this study are based on our cell viability test showing no significant cytotoxicity at levels of 10 µg/mL (Fig. 2), which is comparable to those used for *in vitro* studies (0–50 µg/mL)^32, 51^. The non-cytotoxic concentrations of SWCNTs used herein minimize the confounding factors such as cell death and cell detachment that may influence the uptake and excretion processes, and subsequent quantification of SWCNTs.

Previous studies suggested that SWCNT length might influence SWCNT uptake^52^. A study using DNA-wrapped SWCNTs showed an interesting observation that SWCNTs shorter than 189 (±17) nm are preferentially uptaken by a variety of cells including IMR 90 (primary human lung fibroblasts), A549, MC3T3-E1 and A10, whereas most longer ones remain in cultural supernatants^32^. In comparison with their results, our data seem to be an exception since all lengths of SWCNTs tested in this study were shown to be internalized by macrophages similarly, probably due to their active scavenging behavior for exogenous particles. Our results showed that RAW264.7 cells accumulated SWCNTs in a length-independent but concentration-dependent manner, the efficiency of uptake decreased with increasing concentrations (Fig. 3A). SWCNTs exposed to cells in cell culture are most often associated with serum proteins (such as albumin and globulin) that can be recognized by the numerous receptors expressed on the surface of macrophages^31^ and subsequently are internalized. In addition, SWCNTs can also be internalized by membrane association, diffusion and other cellular pathways^31^. At low concentration of SWCNTs, the receptor mediated uptake could be taken as first order kinetics, similar to that of membrane process^31^. Therefore, by assuming that SWCNTs are internalized by membrane uptake and receptor mediated uptake with first order kinetics, the internalization of SWCNTs could be expressed as following:

1 $${[{\rm{SWCNT}}]}_{{\rm{int}}}=({{\rm{K}}}_{{\rm{mem}}}+{{\rm{K}}}_{{\rm{rec}}}){[{\rm{SWCNT}}]}_{{\rm{ext}}}$$

[SWCNT]_int_ and [SWCNT]_ext_ are the internal and external concentration of SWCNTs, respectively, and K_mem_ represents a superposition of membrane steps, with units of (pg per cell)/(µg mL^−1^ SWCNT), K_rec_ is dependent on receptor concentration. Below 10 µg/mL, we determined that (K_mem_ + K_rec_) = 0.17–0.185 (pg per cell)/(µg mL^−1^ SWCNT) while at higher concentrations (>10 µg/mL), the rate was 0.1–0.13 (pg per cell)/(µg mL^−1^ SWCNT), which is attributed to membrane process and comparable to the 0.1 (pg per cell)/(µg mL^−1^ SWCNT) for SWCNT-PF127 engulfed by J774A.1 cells^31^. Mechanistically, higher concentrations of SWCNTs may saturate receptors that mediate the internalization of SWCNTs, resulting in lower overall uptake efficiency of SWCNTs. Therefore, the K_rec_ was estimated to be 0.05–0.07 (pg per cell)/(µg mL^−1^ SWCNT). These results indicated that, in the process of cellular uptake SWCNTs, membrane process is about two-fold faster than receptor mediated process for our SWCNTs. The observation is in contrast with the SWCNTs-BSA, the receptor process of which is double the membrane association^31^. In comparison of these SWCNTs with different surface chemistry, we learned that our carboxyl group stabilized SWCNTs have comparable membrane association ability with PF127 coated SWCNTs, but 4-fold lower receptor activating activity than BSA coated SWCNTs (K_rec_ = 0.2 pg per cell/(µg mL^−1^ SWCNT))^31^, probably due to following possibility: (1). The difference of cell lines (Raw264.7 vs J774A.1). Although both cells are murine monocytes/macrophages, they display distinct surface protein profiles, for example, Raw264.7 is negative for surface immunoglobulin whereas J774A.1 is in high expression of receptors such as Fc receptor and IgG. That is likely the reason why our SWCNTs showed 4-fold lower receptor uptake; (2). The difference of protein adsorption on SWCNTs. The major component of Fetal bovine serum (FBS) is BSA protein (~62% of total serum protein), but FBS also contain other proteins including globulins (~30%) and a variety of cytokines. This might cause different profiles of adsorption protein on SWCNTs. It has been demonstrated that SWCNTs with different protein coating were not equally bioactive^53^. Therefore, these two types of SWCNTs might display different potent in inducing receptor-mediated internalization; (3). Our carboxylated SWCNTs were briefly mixed with cell culture media, which might lead to much lower protein coating density when compared with SWCNT-BSA that was prepared by co-incubation of SWCNTs and BSA for 2 h with sonication.

Furthermore, SWCNT retention was found to be time-dependent, with the amount of SWCNTs peaking at 8 h and then dropping dramatically afterwards (Fig. 3B). SWCNTs may begin by attaching to the extracellular matrix, which eventually activates the cellular uptake pathways and leads to rapid internalization of SWCNTs. Simultaneously, the interactions also initiate secretion pathways to expel the SWCNTs. This was observed in this study as intracellular SWCNTs decreased but extracellular SWCNTs increased in the supernatant after replacing the exposure medium with fresh medium without SWCNTs (Fig. 4). Therefore, SWCNTs accumulation in cells should be the result of both uptake and excretion processes simultaneously. Our data suggested that S-SWCNTs having slightly higher secretion rate (Fig. 4B). However, we did not find any significant differences in the net accumulation of the three lengths of SWCNT in cells (Fig. 3). It has been reported that healthy cells performing normal, active cellular processes might have internalized some of expelled SWCNTs, increasing their net intracellular SWCNT amount^30^. And by that way, cells retained approximately equal amount of CNTs in the circumstance of continuous exposure. Since the rate of internalization is much faster than rate of excretion at early stage of exposure, as shown in Fig. 4A and B, the concentration of SWCNTs increased until ~8 h, which is in agreement with the findings on the internalization (peaking at ~7 h after recovery) of SWCNTs-BSA by NIH-3T3^30^.

Wang and coworkers showed that BSA coated SWCNTs accumulated continuously within normal rat kidney (NRK) cells across a 3-day period^33^. Our data showed substantial excretion of SWCNTs in 36 h (Fig. 4A,B). Such contrasting results may result from the different cell models used (NRK vs RAW264.7). Moreover, macrophages, which defend hosts against foreign pathogens and particles, might have special active machinery for excreting nanoparticles such as secretory lysosomal pathways^54^. Although several studies have observed excretion of various nanoparticles^52, 54–56^, the detailed mechanisms remain largely unknown and thus worthy of in-depth investigation. Our recent work revealed that that SWCNTs induced activation of P2X_7_ receptor could in turn regulate the exocytosis of SWCNTs, which began with the substantial ATP release, resulting in the opening of P2X_7_R and influx of calcium ions that further induced the activation of PKC and MAPK signaling pathways, pH elevation in lysosomes, reorganization of microtubules, and ultimately the secretion of SWCNTs^57^. P2X7 receptor is highly expressed in macrophages but lower in fibroblasts such as NRK cells, which explains the substantial secretion of SWCNTs in our cells. Also, due to SWCNTs concentration-dependent nature of P2X_7_R activation, continuous SWCNTs exposure tends to stimulate stronger and longer P2X_7_R activation and thus exocytosis of more SWCNTs, whereas removing SWCNTs exposure inevitably minimized the excitation of exocytosis pathway, which might explain the minimal difference in intracellular L-SWCNT amount in cells under either continuous or transitional SWCNTs exposure (Fig. 3B vs Fig. 4B).

Results from the current and previous studies suggest that SWCNTs could be readily internalized in macrophages^20, 58^. It has been revealed that the internalization mechanisms of CNTs varied with the degree of dispersion, formation of supramolecular complexes and nanotube length^59^. For individually dispersed CNTs with length less than 1 µm, passive diffusion and receptor mediated endocytosis (RME) are responsible for the uptake of CNTs. The energy-independent diffusion of SWCNTs was confirmed by our results showing that NaN_3_ could inhibit the uptake of 75–78% SWCNTs, which means endocytosis as the main internalization mechanism for the uptake of SWCNTs. Since there are four types of endocytosis: clathrin-mediated, caveolae-mediated, macropinocytosis, and phagocytosis^60^, the relative contribution of these endocytic pathways to the uptake of SWCNTs is seldom resolved. Phagocytosis is mediated through receptor (such as mannose receptor, Fcγ receptor and scavenger receptor) binding and responsible for the internalization of particulate matters larger than 1 µm (with a maximum phagocytosis rate of 2–3 µm) in macrophages^61^. Although the higher end of L-SWCNT length distribution might fall into the phagocytosis range, most of the SWCNTs should have been internalized through other pathways. This was confirmed by our result showing that amiloride, an inhibitor specific for macropinocytosis, inhibited over 90% of SWCNT internalization (Fig. 5 and Table 2), which also suggested that SWCNTs were internalized mainly through macropinocytosis. Literatures are replenished with the internalization mechanism of SWCNTs involving RME, but all CNTs were functionalized with DNA^52^ or proteins^46, 50^ that might activate receptors on the cell surface. Here we reported that macropinocytosis was the major mechanism responsible for the internalization of SWCNTs without biomolecules coating. The observation is consistent with our TEM results showing that most SWCNTs present in large vesicles (0.2–5 µm), which are typically formed during macropinocytosis.

Clathrin-dependent endocytosis is a receptor-mediated endocytosis (RME)^62, 63^. Nanoparticles less than 200 nm (optimal size is ~25 nm) are usually deposited in the vesicles^64, 65^. In this study, clathrin-mediated endocytosis was clearly length selective, as CPM treatment decreased SWCNT internalization with decreasing SWCNT length (Fig. 5D), suggesting that clathrin-mediated endocytosis, although a minor pathway, was relatively important for internalizing shorter SWCNTs, which is consistent with the results showing that 100–200 nm long SWCNTs (chitosan-coated) are internalized in cells mainly through clathrin-dependent pathways^66^. Finally, we observed endocytosis of SWCNTs was also significantly inhibited by MβCD (Table 2), suggesting that no single pathway is fully responsible for internalizing SWCNTs. For energy-dependent endocytosis, our SWCNTs were internalized by the pathways in the order of macropinocytosis> caveolae-mediated endocytosis> clathrin-dependent endocytosis. The conclusion is in accordance with the one obtained for titanium dioxide nanoparticles in human prostate cancer cells^67^ and consolidates our previous assumption that SWCNT uptake is the result of both membrane uptake and receptor-mediated processes (equation 1), which also explains the contrasting observations that SWCNTs may enter cells in an energy-independent way^68^ and by endocytosis, which is energy-dependent^69^.

In summary, we quantitatively measured the intracellular retention of three SWCNTs with different lengths (195 (±63), 390 (±50), and 630 (±171) nm) in macrophages to understand the uptake and exocytosis processes and to resolve the relative contribution of uptake mechanisms during SWCNT internalization. The results showed that the net cellular accumulation of SWCNTs was length-independent when the length of SWCNTs is less than 1 μm and varied with exposure time, with the intracellular SWCNTs peaked at 8 h, which is followed by rapid excretion. S-SWCNTs were expulsed faster than L- and M-SWCNTs. The uptake rate of SWCNTs decreased with increasing concentration, suggesting that SWCNTs accumulation in cells is a saturable process. The internalization mechanisms of SWCNTs is mostly energy dependent and involves several pathways, in the order of macropinocytosis > caveolae-mediated endocytosis > clathrin-dependent endocytosis. Clathrin-mediated endocytosis is relatively important for internalizing shorter SWCNTs. A better understanding of the processes and mechanisms of uptake and exocytosis of SWCNTs may facilitate the assessment of CNT risk on health and environment and the design of CNT products with improved functionality and lower toxicity.

## Methods

### Materials and reagents

The murine monocytic RAW264.7 cells (ATCC: TIB-71) were acquired from American Type Culture Collection (Manassas, VA). All ingredients for cell culture media were purchased from Gibco (Invitrogen, Paisley, UK). SWCNTs in high purity (CNT purity > 95%, SWCNT purity > 90%, ash  < 5 wt%) synthesized by chemical vapor deposition were obtained from Chengdu Organic Chemicals Co., Ltd (Sichuan, China). Further information can be found on the company website: http://www.timesnano.com/. CPM, MβCD, NaN_3_, and amiloride were purchased from Sigma-Aldrich (St. Louis, USA). WST-1 was purchased from Roche (Penzberg, Germany).

### Preparation and characterization of different lengths of SWCNTs

SWCNTs were prepared according to the procedure described previously^20^. Three different lengths of SWCNTs were obtained by controlling the oxidation time. Briefly, 10 mg of SWCNTs were suspended in 40 mL of a 3:1 mixture of concentrated H_2_SO_4_/HNO_3_ in a 200-mL flask and sonicated in a water bath (KQ-500DV, 40 kHz) at 40–50 °C for 24 h to obtain L-SWCNTs, 36 h for M-SWCNTs, and 48 h for S-SWCNTs. The resulting suspensions were then diluted with 200 mL of deionized water and filtered through a membrane (pore size 0.22 μm) before washing with 50 mL of deionized water on the membrane. The SWCNTs were re-suspended in sterilized deionized water at a concentration of 1 mg/mL by sonication for 5 min (KQ-500DV, 40 kHz). The SWCNT suspensions were black and well dispersed with neutral pH.

The zeta-potential and hydrodynamic diameters of SWCNTs were measured by using a Zetasizer Nano (Malvern Instruments, Malvern, UK). Samples were diluted to a concentration 20 μg/mL in deionized water or culture medium. The morphology and length of SWCNTs were examined using a HitachiH-7500 TEM (Tokyo, Japan). Specifically, the samples were diluted to 0.05 mg/mL in isopropanol solution and then centrifuged at 3000 g for 30 min to remove amorphous carbon and other impurities. The supernatants were then collected, re-suspended in isopropanol solution, and sonicated in a water bath for 5 min (KQ-500DV, 40 kHz). The above procedure was repeated five times to obtain a final suspension that was precipitated onto a copper net and dried at room temperature. The resulting TEM images were used to determine the length distribution of SWCNTs with Image-Pro Plus 6.0 (Media Cybernetics, Inc., Bethesda, MD, USA). More than 100 SWCNTs from representative TEM images were measured for each type of SWCNTs. The infrared spectra of SWCNTs were detected using a FT-IR spectrometer (JASCO, Inc., Easton, MD, USA). Raman measurements were performed on an in Via Raman spectroscopy (Renishaw Plc., Gloucestershire, UK) with excitation wavelength at 532 nm. An Agilent 7500 inductively coupled plasma mass spectrometer (ICP-MS) (Santa Clara, CA, USA) was used to measure the metal content of SWCNTs.

### Cell culture and treatments

RAW264.7 cells were cultured in complete RPMI1640 (c-RPMI) medium consisting of RPMI-1640 and 10% heat-deactivated fetal bovine serum supplemented with 20 mM L-glutamine and 100 UmL^−1^ penicillin/streptomycin, at 37 °C, 5% CO_2_/95% air, and 95% humidity. For all quantification experiments, cells were seeded in 6-well plates at a density of 5 × 10^5^ cells/mL and allowed to attach for overnight. Stock solution of 1 mg/mL SWCNTs was diluted in c-RPMI to desired concentrations for cell exposure.

### BCA assay for protein quantification

The total cellular protein was determined by using a microplate BCA protein assay kit (KangWei, China). Aqueous BSA standards were prepared from a 2 mg/mL BSA stock solution by serial dilutions. Ten microliters of diluted cell lysate samples or BSA standards were dispensed into a 96-well plate and then 200 μL of the BCA reagent were added. The mixture was incubated at 37 °C for 30 min before reading the absorbance at 595 nm on a Thermo Varioskan Flash microplate reader (Winooski, VT, USA). The protein concentration of each sample was determined by plotting against the standard curve.

### SDS-PAGE gel electrophoresis for SWCNTs quantification

After incubation, cells were washed twice with PBS to remove extra SWCNTs unattached to cells. The cells were lysed with 160 μL lysis buffer consisting of 1% SDS, 1 mM MgCl_2_, and 1 mM CaCl_2_ for 3 min. The cell lysates were then sonicated with a cell disruptor to reduce the viscosity of the solution. SDS-PAGE gel electrophoresis was performed using a standard BD Mini Vertical Gel (10 cm × 8 cm) with 10 loading wells. We used 4% stacking gel as the loading substrate for SWCNTs. Twenty microliters of the cell lysate samples were loaded onto the SDS-PAGE gel and electrophoresed at 120 V for 2 h. The loading wells were then sealed with 4% stacking gel for 30 min, after then the gel was scanned by using a UMAX scanner. The integrated optical densitometry (IOD) of SWCNT band on the gel image was quantified using Gelpro software (version 4.0). When performing SDS-PAGE, we controlled the volume of cell lysates applied to each well so that the total amount of protein remained the same.

### Determination of SWCNT exocytosis by UV-vis-NIR spectroscopy and SDS-PAGE analysis

Cells were treated with or without 10 µg/mL of SWCNTs for 5 h and then washed three times with PBS and further incubated with fresh c-RPMI (without SWCNTs). The supernatant was collected for UV-vis-NIR analyses at indicated time points. Meanwhile, intracellular SWCNT amount of cells in corresponding wells were analyzed via SDS-PAGE gel electrophoresis as mentioned above.

### Endocytosis inhibition analysis

To explore underlying mechanisms of cellular uptake of SWCNTs, RAW264.7 cells were pre-incubated with four specific endocytosis inhibitors (10 µg/mL CPM, 10 mM MβCD, 30 mM NaN_3_, and 50 µg/mL Amiloride) in c-RPMI for 4 h, separately. After removal of inhibitor solutions, cells were washed twice with PBS and then incubated with 10 µg/mL of L-, M-, or S-SWCNTs for another 5 h. The cells without inhibitor treatment were used as control. The amount of intracellular SWCNTs in cells from treatment and control groups was measured via SDS-PAGE method.

### Ultrathin section of RAW264.7 cells for SWCNTs examination and scanning electron microscopy (SEM)

SWCNT distribution within RAW264.7 cells was investigated using TEM. Cells at a concentration of 2 × 10^6^ were exposed to SWCNTs for 24 h and collected, washed twice with PBS, and pelleted by centrifugation at 350 g for 10 min at 4 °C. The cells were then pre-fixed with 2.5% glutaraldehyde in PBS for 10 min at room temperature and stored at 4 °C overnight. Cells were post-fixed with 1% osmium tetroxide at room temperature for 2 h, followed by dehydration and resin embedding. Ultrathin sections of macrophages were cut using a Leica EMUC6 ultramicrotome (Wetzlar, Germany) and placed on 200-mesh carbon-coated copper grids. Samples were stained with uranyl acetate and lead citrate and observed with a FEI TENAI Sprit TEM (Eindhoven, Noord-Brabant, Netherlands) at 80 kV. For SEM imaging, cells grown on sterilized coverslips were exposed to L-SWCNTs (10 µg/mL) for 24 h and then fixed with 2.5% glutaraldehyde in PBS for overnight. Next, cells were washed with PBS twice, followed by wash with pure ethanol (100%) for 10 min and dried. After coating with platinum, the coverslips were observed on a Hitachi SU8000 SEM (Hitachi, Tokyo, Japan).

### Labeling of SWCNTs for tracking SWCNTs within Cells

To determine whether SWCNTs within macrophages were localized in lysosomes or not, SWCNTs was covalently labeled with an amine-containing fluorescent dye, Alexa Fluor 488, through EDC (1-ethyl-3-[3-dimethylaminopropyl] carbodiimide hydrochloride)/NHS (N-hydroxysulfosuccinimide) mediation in MES buffer. The labeled SWCNTs were further purified and concentrated by centrifugation at 12,000 rpm in ultrafiltration tubes (Millipore, Germany) and then diluted to desired concentration. After 24 h exposure, cells was washed with PBS and then labeled with Lysotracker Red DND-99 (200 nM) for 30 min according to the protocol provided by manufacturer (Invitrogen, USA). The co-localization of SWCNTs and lysosomes were performed via confocal laser scanning microscopy (Leica, Mannheim, Germany).

### Cell viability assay

Two mL of cells were seeded in a 6-well plate at a density of 5 × 10^5^/mL and allowed to attach for overnight. After that, cells were exposed to different concentrations of SWCNTs (control, 10, 20 µg/mL) for 24 h, or 10 µg/mL for different time (0, 5, 8, 12, 24, 36 h). In control group, cells were exposed to the same amount of dilution water as that of treatment. After exposure, cells (with confluency 80–90%) were washed with PBS and incubated in c-RPMI containing 10% (v/v) WST-1 solution (Roche, Mannheim, Germany) for 1 h in cell incubator. The absorbance of each well was measured at 450 nm using a Thermo Varioskan Flash microplate reader.

### Statistical analysis

The data were expressed as the mean ± SEM, and the difference between groups was evaluated using Student’s *t*-test, with the significance level set at *p < 0.05 or **p < 0.01.

## Electronic supplementary material

Supplementary Information

## References

[1] Warheit DB (2006). What is currently known about the health risks related to carbon nanotube exposures?. Carbon.

[2] Zhao, Q. Q., Boxman, A. & Chowdhry, U. Nanotechnology in the chemical industry– opportunities and challenges. *J. Nanopart. Res*. **5**, 567–572 (2003).

[3] Liang F, Chen B (2010). A Review on Biomedical Applications of Single-Walled Carbon Nanotubes. Curr. Med. Chem..

[4] Klumpp C, Kostarelos K, Prato M, Bianco A (2006). Functionalized carbon nanotubes as emerging nanovectors for the delivery of therapeutics. Biochim. Biophys. Acta..

[5] Yang, R. *et al*. Single-walled carbon nanotubes-mediated *in vivo* and *in vitro* delivery of siRNA into antigen-presenting cells. *Gene Thera*. **13** (2006). 10.1038/sj.gt.3302808 16838032

[6] Pantarotto D (2004). Functionalized carbon nanotubes for plasmid DNA gene delivery. Angew. Chem. Int. Edit..

[7] Milne WI (2008). Carbon Nanotubes. E-Nano Newsletter.

[8] Braakhuis, H. M. *et al*. Particle size dependent deposition and pulmonary inflammation after short-term inhalation of silver nanoparticles. *Part. Fibre Toxicol*. **11**, doi:10.1186/s12989-014-0049-1 (2014). 10.1186/s12989-014-0049-1 PMC4410796 25227272

[9] Yang ST (2008). Long-term accumulation and low toxicity of single-walled carbon nanotubes in intravenously exposed mice. Toxicol. Lett..

[10] Wang H (2004). Biodistribution of Carbon Single-Wall Carbon Nanotubes in Mice. J. Nanosci. Nanotechnol..

[11] Luisa Campagnolo, G. P. *et al*. Biodistribution and toxicity of pegylated single walled carbon nanotubes in pregnant mice. *Part. Fibre Toxicol*. **10**, doi:10.1186/1743-8977-10-21 (2013). 10.1186/1743-8977-10-21 PMC3679973 23742083

[12] Lam CW, James JT, McCluskey R, Hunter RL (2004). Pulmonary toxicity of single-wall carbon nanotubes in mice 7 and 90 days after intratracheal instillation. Toxicol. Sci..

[13] Shvedova AA (2005). Unusual inflammatory and fibrogenic pulmonary responses to single-walled carbon nanotubes in mice. Am. J. Physiol. Lung Cell. Mol. Physiol..

[14] Shvedova AA (2008). Inhalation vs. aspiration of single-walled carbon nanotubes in C57BL/6 mice: inflammation, fibrosis, oxidative stress, and mutagenesis. Am. J. Physiol. Lung Cell. Mol. Physiol..

[15] Li Z (2004). Pulmonary carbon nanotube exposure and oxidative status in vascular system. Free Radical Bio. Med..

[16] Li Z (2007). Cardiovascular effects of pulmonary exposure to single-wall carbon nanotubes. Environ. Health Persp..

[17] Nygaard UC (2009). Single-Walled and Multi-Walled Carbon Nanotubes Promote Allergic Immune Responses in Mice. Toxicol. Sci..

[18] Mangum JB (2006). Single-Walled Carbon Nanotube (SWCNT)-induced interstitial fibrosis in the lungs of rats is associated with increased levels of PDGF mRNA and the formation of unique intercellular carbon structures that bridge alveolar macrophages *In Situ*. Part. Fibre Toxicol..

[19] Van Berlo D (2014). Apoptotic, inflammatory, and fibrogenic effects of two different types of multi-walled carbon nanotubes in mouse lung. Arch. Toxicol..

[20] Dong PX, Wan B, Guo LH (2012). *In vitro* toxicity of acid-functionalized single-walled carbon nanotubes: effects on murine macrophages and gene expression profiling. Nanotoxicology.

[21] Dong PX (2013). Exposure of single-walled carbon nanotubes impairs the functions of primarily cultured murine peritoneal macrophages. Nanotoxicology.

[22] Oberdorster G (2005). Principles for characterizing the potential human health effects from exposure to nanomaterials: elements of a screening strategy. Part. Fibre Toxicol..

[23] Poland CA (2008). Carbon nanotubes introduced into the abdominal cavity of mice show asbestos-like pathogenicity in a pilot study. Nat. Nanotechnol..

[24] Liu J (2012). Influence of surface functionalization and particle size on the aggregation kinetics of engineered nanoparticles. Chemosphere.

[25] Manke A (2014). Effect of Fiber Length on Carbon Nanotube-Induced Fibrogenesis. Int. J. Mol. Sci..

[26] Dorger M (2001). Differential responses of rat alveolar and peritoneal macrophages to man-made vitreous fibers *in vitro*. Environ. Res..

[27] Shi X (2011). Cell entry of one-dimensional nanomaterials occurs by tip recognition and rotation. Nat. Nanotechnol..

[28] Murphy FA (2011). Length-Dependent Retention of Carbon Nanotubes in the Pleural Space of Mice Initiates Sustained Inflammation and Progressive Fibrosis on the Parietal Pleura. Am. J. Pathol..

[29] Vietti G (2013). Towards predicting the lung fibrogenic activity of nanomaterials: experimental validation of an *in vitro* fibroblast proliferation assay. Part. Fibre Toxicol..

[30] Holt BD, Dahl KN, Islam MF (2012). Cells Take up and Recover from Protein-Stabilized Single-Wall Carbon Nanotubes with Two Distinct Rates. ACS Nano.

[31] Boyer PD, Holt BD, Islam MF, Dahl KN (2013). Decoding membrane- versus receptor-mediated delivery of single-walled carbon nanotubes into macrophages using modifications of nanotube surface coatings and cell activity. Soft Matter.

[32] Becker ML (2007). Length-dependent uptake of DNA-wrapped single-walled carbon nanotubes. Adv. Mater..

[33] Wang RH (2009). Gel Electrophoresis Method to Measure the Concentration of Single-Walled Carbon Nanotubes Extracted from Biological Tissue. Anal. Chem..

[34] Song MY (2014). Co-exposure of Carboxyl-Functionalized Single-Walled Carbon Nanotubes and 17 alpha-Ethinylestradiol in Cultured Cells: Effects on Bioactivity and Cytotoxicity. Environ. Sci. Technol..

[35] Deloid GM (2015). Advanced computational modeling for *in vitro* nanomaterial dosimetry. Part. Fibre Toxicol..

[36] Cherukuri P, Bachilo SM, Litovsky SH, Weisman RB (2004). Near-infrared fluorescence microscopy of single-walled carbon nanotubes in phagocytic cells. J. Am. Chem. Soc..

[37] Andon FT (2013). Biodegradation of Single-Walled Carbon Nanotubes by Eosinophil Peroxidase. Small.

[38] Wang LH, Rothberg KG, Anderson RG (1993). Mis-assembly of clathrin lattices on endosomes reveals a regulatory switch for coated pit formation. J. Cell Biol..

[39] Huang J (2012). Mechanism of cellular uptake of graphene oxide studied by surface-enhanced Raman spectroscopy. Small.

[40] Koivusalo M (2010). Amiloride inhibits macropinocytosis by lowering submembranous pH and preventing Rac1 and Cdc42 signaling. J. Cell Biol..

[41] Sayes CM (2006). Functionalization density dependence of single-walled carbon nanotubes cytotoxicity *in vitro*. Toxicol. Lett..

[42] Ramanathan T, Fisher FT, Ruoff RS, Brinson LC (2005). Amino-Functionalized Carbon Nanotubes for Binding to Polymers and Biological Systems. Chem. Mater..

[43] Pan BF (2007). Cellular Uptake Enhancement of Polyamidoamine Dendrimer Modified Single Walled Carbon Nanotubes. J. Biomed. Pharm. Eng.

[44] Yang ST (2008). Covalently PEGylated carbon nanotubes with stealth character *in vivo*. Small.

[45] Kagan VE (2010). Carbon nanotubes degraded by neutrophil myeloperoxidase induce less pulmonary inflammation. Nat Nanotechnol..

[46] Liu J (1998). Fullerene Pipes. Science.

[47] Smart SK, Cassady AI, Lu GQ, Martin DJ (2006). The biocompatibility of carbon nanotubes. Carbon.

[48] Shi Kam NW, Dai H (2005). Carbon Nanotubes as Intracellular Protein Transporters: Generality and Biological Functionality. J. Am. Chem. Soc..

[49] Villa CH (2008). Synthesis and Biodistribution of Oligonucleotide-Functionalized Tumor-Targetable Carbon Nanotubes. Nano Lett..

[50] Shi Kam NW, Jessop TC, Wender PA, Dai H (2004). Nanotube molecular transporters: internalization of carbon nanotube-protein conjugates into Mammalian cells. J. Am. Chem. Soc..

[51] Rodríguez-Yáñez Y (2015). Commercial single-walled carbon nanotubes effects in fibrinolysis of human umbilical vein endothelial cells. Toxicol. in Vitro.

[52] Jin H, Heller DA, Sharma R, Strano MS (2009). Size-dependent cellular uptake and expulsion of single-walled carbon nanotubes: single particle tracking and a generic uptake model for nanoparticles. ACS Nano.

[53] Brian D. H (2012). Not all protein-mediated single-wall carbon nanotube dispersions are equally bioactive. Nanoscale.

[54] Oh N, Park JH (2014). Endocytosis and exocytosis of nanoparticles in mammalian cells. Int. J. Nanomed..

[55] Chen R, Huang G, Ke PC (2010). Calcium-enhanced exocytosis of gold nanoparticles. Appl. Phys. Lett..

[56] Jin H, Heller DA, Michael S (2008). Strano. Single-Particle Tracking of Endocytosis and exocytosis of single walled carbon nanotubes in NIH-3T3 cell. Nano Lett..

[57] Cui XJ (2016). Crucial Role of P2X7 Receptor in Regulating Exocytosis of Single-Walled Carbon Nanotubes in Macrophages. Small.

[58] Wan B (2013). Single-walled carbon nanotubes and graphene oxides induce autophagosome accumulation and lysosome impairment in primarily cultured murine peritoneal macrophages. Toxicol. Lett..

[59] Raffa V (2010). Physicochemical properties affecting cellular uptake of carbon nanotubes. Nanomedicine.

[60] Rupper A, Cardelli J (2001). Regulation of phagocytosis and endo-phagosomal trafficking pathways in Dictyostelium discoideum. BBA. Gen. Subjects.

[61] Xiao Y (2010). Dynamics and mechanisms of quantum dot nanoparticle cellular uptake. J. Nanobiotechnol..

[62] Howe CL (2005). Modeling the signaling endosome hypothesis: Why a drive to the nucleus is better than a random walk. Faseb J..

[63] Kholodenko BN (2003). Four-dimensional organization of protein kinase signaling cascades: the roles of diffusion, endocytosis and molecular motors. J. Exp. Biol..

[64] Prokop A, Davidson JM (2008). Nanovehicular intracellular delivery systems. J. Pharm. Sci..

[65] Liu, B. R. *et al*. Endocytic Trafficking of Nanoparticles Delivered by Cell-penetrating Peptides Comprised of Nona-arginine and a Penetration Accelerating Sequence. *Plos One* **8**, e67100, doi:10.1371/journal.pone.0067100 (2013). 10.1371/journal.pone.0067100 PMC3694042 23840594

[66] Kang B (2010). Cell response to carbon nanotubes: size-dependent intracellular uptake mechanism and subcellular fate. Small.

[67] Thurn KT (2011). Endocytosis of titanium dioxide nanoparticles in prostate cancer PC-3M cells. Nanomed. Nanotechnol..

[68] Kostarelos K (2007). Cellular uptake of functionalized carbon nanotubes is independent of functional group and cell type. Nat. Nanotechnol..

[69] Yaron PN (2011). Single wall carbon nanotubes enter cells by endocytosis and not membrane penetration. J. Nanobiotechnol..

