# Supplementary material for: Length effects on the dynamic process of cellular uptake and exocytosis of single-walled carbon nanotubes in murine macrophage cells
Source: Sci Rep. 2017 May 10;7:1518. doi: 10.1038/s41598-017-01746-9 (PMC5431871; doi:10.1038/s41598-017-01746-9)
Supplement: Supplementary file 1 — Supplementary Information [file 41598_2017_1746_MOESM1_ESM.pdf]

Length effects on the dynamic process of cellular uptake and exocytosis of single-walled carbon nanotubes in murine macrophage cells

Xuejing Cui,[1,2] Bin Wan,[1,2,*] Yu Yang,[1] Xiaomin Ren,[1] Liang-Hong Guo[1,2,3,*]

*Corresponding authors: Dr. Bin Wan and Dr. Liang-Hong Guo

Email: binwan@rcees.ac.cn, LHGuo@rcees.ac.cn

First author: Xuejing Cui

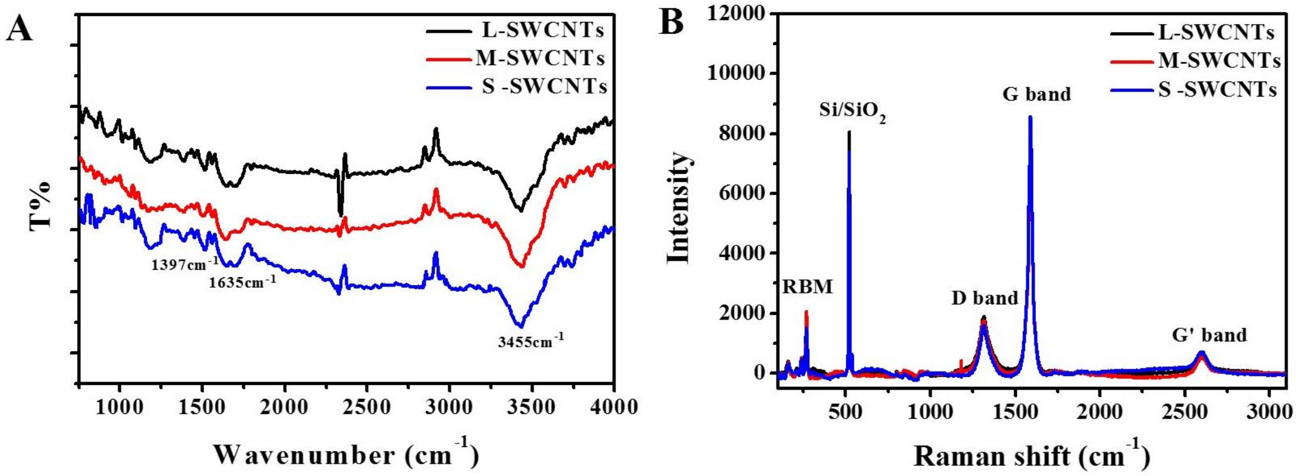

Supplementary Figure S1. Characterization of SWCNTs. (A) FT-IR spectra of SWCNTs. (B) Typical Raman spectra of SWCNTs.

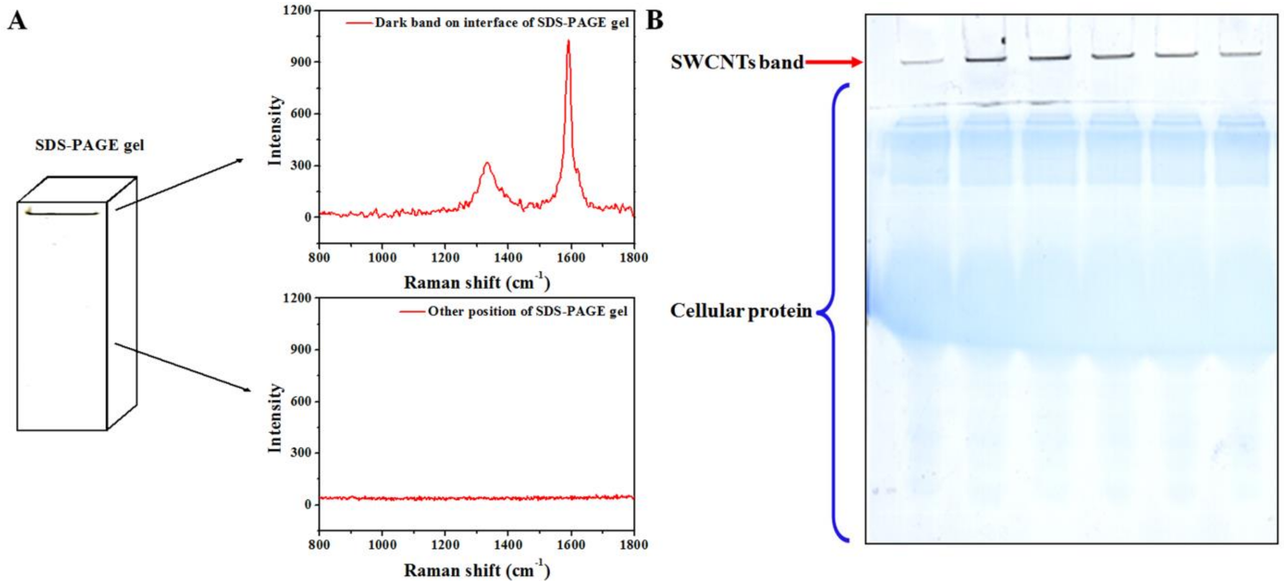

Supplementary Figure S2. Raman detection of SWCNTs and Coomassie blue staining. (A) Raman spectroscopic analysis of an SDS-PAGE gel loaded with 20 μL of cell lysates after exposure to 10 μg/mL SWCNTs following electrophoresis at 120 V for 2 h. Raman spectra were acquired from various positions (Y axis 20 points, z axis 50 points, total 1000 points) on and below the interface of the stacking gel and the loading well. The plot of integrated Raman intensities from 1300 cm^{-1} to 2000 cm^{-1}, representing the D-band (1347 cm^{-1}) and G-band (1594 cm^{-1}) resonance of SWCNTs. Up-panel represented D- and G-resonance of the dark band in the interface. Lower-panel showed no D- and G-resonance of SWCNTs which indicated that SWCNTs were not found elsewhere, which verified the reliability of SDS-PAGE method. (B) Coomassie blue staining of the gel showing that SWCNTs were deposited in loading wells and were separated from cellular proteins.

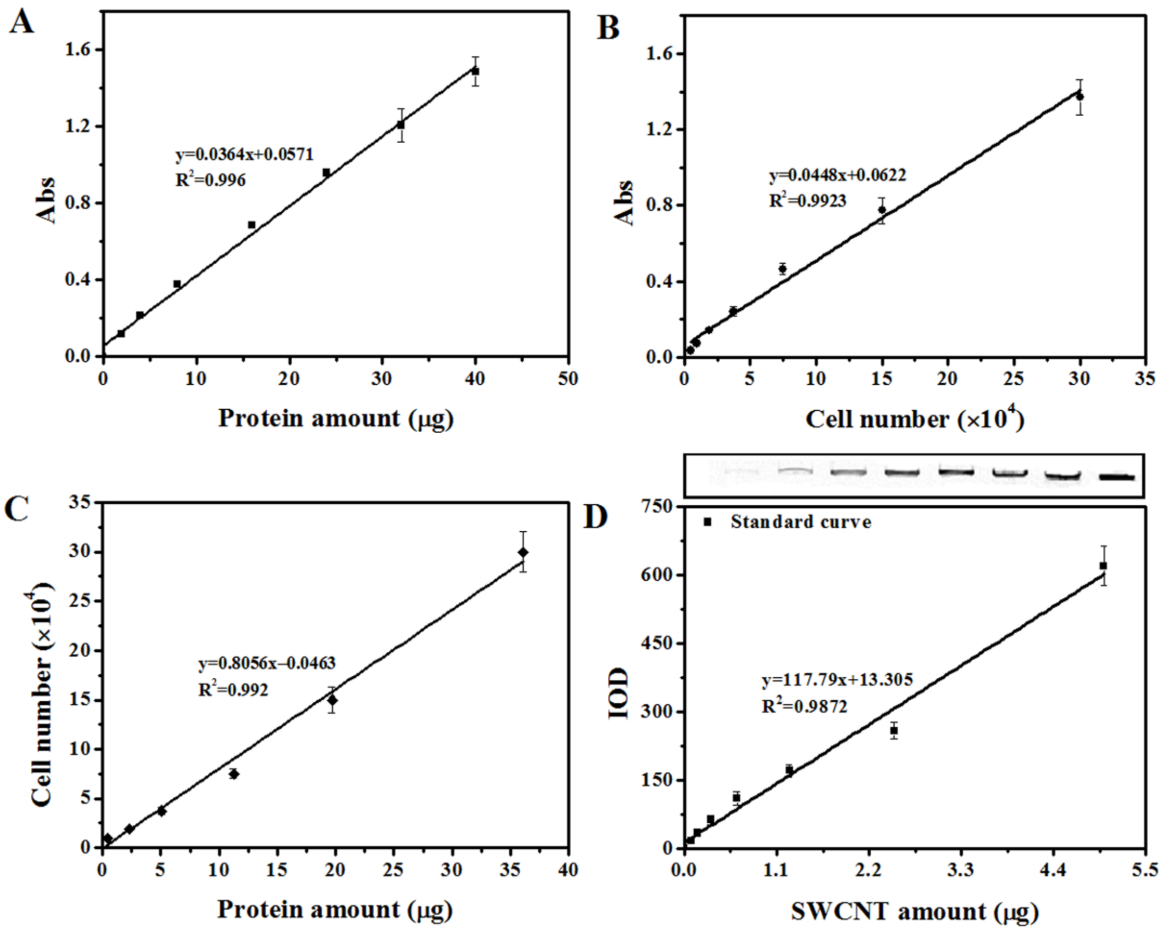

Supplementary Figure S3. A series of standard curves for quantifying SWCNTs amount per cell. (A) Standard curve of protein amount and absorbance (Abs), as measured by BCA method. (B) Standard curve of cell number and absorbance. (C) Standard curve of protein amount and cell number (R^2 = 0.992).（D）A series of concentrations of SWCNTs (total volume was 20 µL for each well) were loaded into wells. IOD (intensity of dark band) intensity is proportional to the amount of SWCNTs in each well (R^2 = 0.9872). Each data point is the mean of three individual experiments. Error bars represent SEM. Up-panel showed corresponding representative SWCNTs bands in the gel image. Data are presented as mean ± standard error of mean.

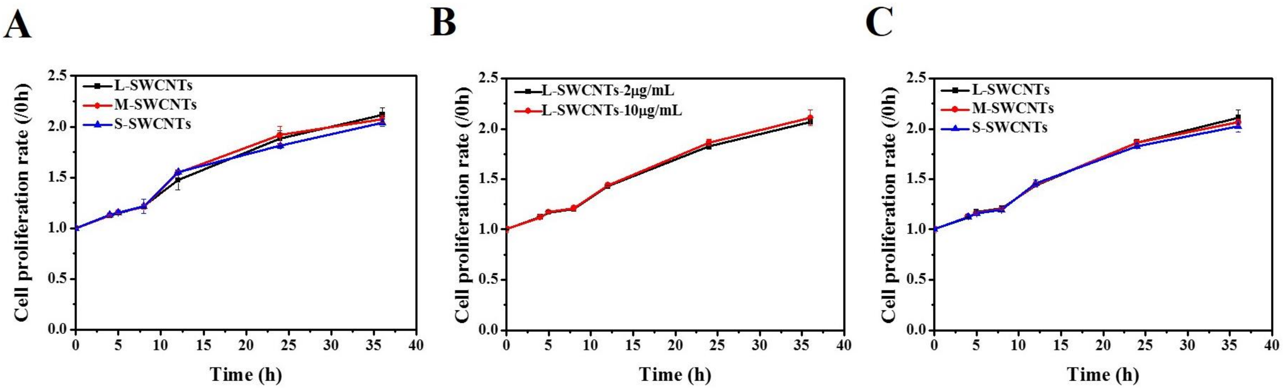

Supplementary Figure S4. The cell proliferation over time, as determined by an automatic cell counter (Countess, Invitrogen). (A) The proliferation rate of cell treated with L, M, and S-SWCNTs (10 μg/mL) for different time period. (B) The proliferation rate of cell treated with L-SWCNTs (2 and 10 μg/mL) for different time period after replacing SWCNTs exposure solutions with fresh medium at 5 h. (C) The proliferation rate of cell treated with L, M, and S-SWCNTs (10 μg/mL) for different time period after replacing SWCNTs exposure solutions with fresh medium at 5 h. After treatment, the cells (10 μL) were mixed with trypan blue reagent (10 μL) for 1 min, and the cell number were then measured by automatic cell counter (Countess, Invitrogen).

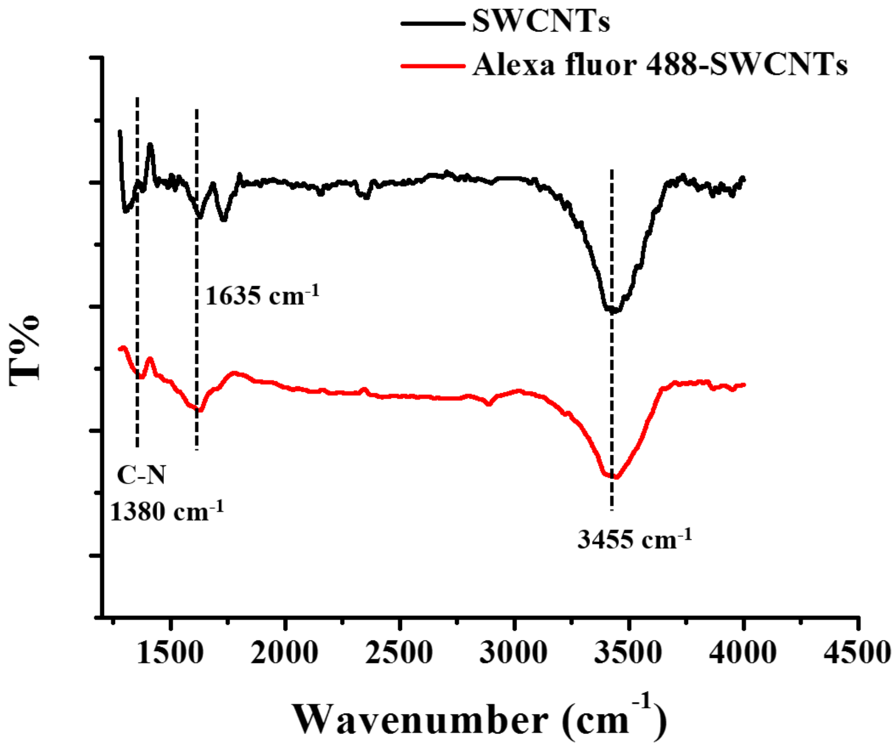

Supplementary Figure S5. FT-IR spectra of SWCNTs and Alexa fluor 488 conjugated SWCNTs.

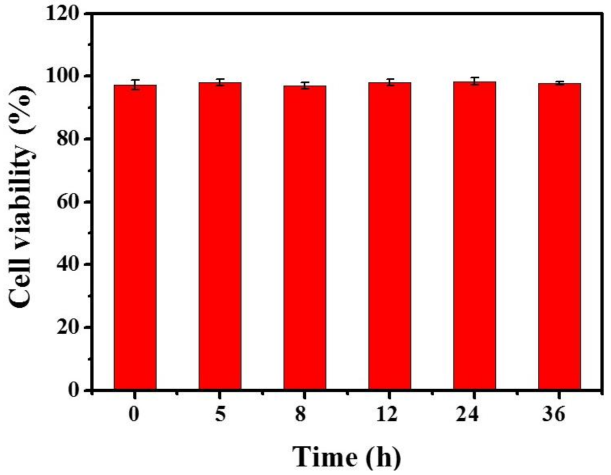

Supplementary Figure S6. The viability of cells treated with 10 µg/mL L-SWCNTs at different time points after removing exposure solutions at 5 h, as determined by an automatic cell counter (Countess, Invitrogen). Data are presented as mean ± standard error of mean.

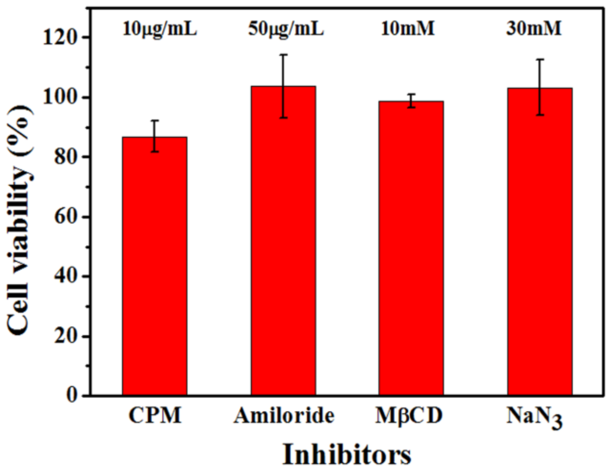

Supplementary Figure S7. The viability (Treatment/control) of cells treated with inhibitors for 4 h, as determined by WST-1 assay. Data are presented as mean ± standard error of mean.
